# L-Rhamnose Is Often an Important Part of Immunodominant Epitope for Pneumococcal Serotype 23F Polysaccharide Antibodies in Human Sera Immunized with PPV23

**DOI:** 10.1371/journal.pone.0083810

**Published:** 2013-12-31

**Authors:** Saeyoung Park, Moon H. Nahm

**Affiliations:** University of Alabama at Birmingham, Birmingham, Alabama, United States of America; Instituto Butantan, Brazil

## Abstract

*Streptococcus pneumoniae* is a major human pathogen which expresses more than 90 serologically distinct capsular polysaccharides (PS) on the surface. Since pneumococcal PSs elicit protective antibodies against pneumococcal diseases, it is important to identify the immunological epitope eliciting anti-pneumococcal PS antibodies. L-rhamnose is a part of the 23F PS repeating unit and is known to be a critical part of immunodominant epitope which elicits antibodies against pneumococcal serotype 23F PS. In order to determine if L-rhamnose is a part of epitope recognized by functional antibodies specific for serotype 23F PS in human serum samples, we evaluated the opsonophagocytic killing of serotype 23F pneumococci by serum antibodies specific for L-rhamnose. Using 10 mM L-rhamnose, opsonic capacities (opsonic indices) of serum antibodies were inhibited by 60% in 19 sera (36%) and 30–60% in 16 sera (30%) out of 53 sera from young and old adults immunized with 23-valent pneumococcal polysaccharide vaccine (PPV23). Interestingly, when IgM antibodies were depleted from immune sera in order to preferentially study IgG antibodies, the proportion of young adult sera showing more than 60% inhibition in opsonic capacity by 10 mM of L-rhamnose increased from 33% (11/31) to 68% (21/31). On the other hand, IgM depletion did not alter the proportion for old adult sera. Therefore, young and old adults may produce different antigen binding profiles of IgG antibodies against serotype 23F PS.

## Introduction

*Streptococcus pneumoniae* is a significant pathogenic bacterium that causes several diseases such as pneumonia, bacteremia, meningitis, and otitis media [1]. Among many kinds of surface molecules on pneumococci, capsular polysaccharide (PS) is one of the major virulence factors [2]. Pneumococcal capsular PS is a polymer of carbohydrate repeating units, and so far at least 93 different serotypes are described based on the structure of PS [3]–[6]. Since antibodies against pneumococcal PS are highly protective, adults are immunized with a 23-valent pneumococcal PS vaccine (PPV23) and young children are immunized with several pneumococcal conjugate vaccines (PCVs), which are produced by conjugating 7–13 different capsular PS to a carrier protein [7]–[11].

**Figure 1:**
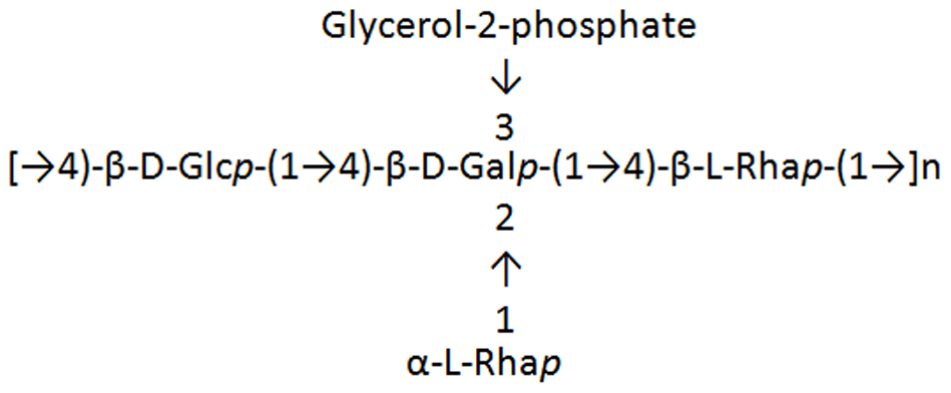
Structure of the PS of *Streptococcus pneumoniae* serotype 23F.

With increasing numbers of conjugates in a vaccine, the complexity of a vaccine greatly increases, and thus there is a need to search for a simple epitope that can elicit antibodies against pneumococci [12]–[15]. Serotype 23F capsular PS has tetrasaccharide repeating units containing one glucose, one galactose and two L-rhamnose residues (Figure 1). One of the two rhamnose residues forms a branch of the backbone (Figure 1) [16], [17] and previous studies showed that the structures including branched L-rhamnose is the dominant epitope recognized by horse and rabbit antisera [16], [18]. However, it has not yet been determined whether antibodies targeting rhamnose are functional (i.e., opsonic). If functional, it is unclear whether rhamnose-specific antibodies remain functional among old adults, inasmuch as antibodies from old adults tend to be poorly functional [16], [18].

In this study, therefore, we used an opsonophagocytosis assay to determine if L-rhamnose-containing structure is a functional epitope of human antibodies specific for pneumococcal serotype 23F PS from a large number of serum samples obtained from young or old adults immunized with PPV23. We studied these rhamnose specific antibodies to investigate the functional difference in the anti-23F antibody repertoire between young and old adults.

## Materials and Methods

### Serum samples

Two groups of anonymous human sera were used. One group was sera from old adults (70–79 years of age; N = 44), who had received one PPV23 at least 5 years prior to enrollment. They were immunized with 0.5 ml of pneumococcal PS vaccine (PPV23) (Pneumovax®, Merck, Whitehouse Station NJ) one month before phlebotomy [19]. Pre-immune sera were obtained before enrollment vaccination, and analyzed for functional antibody activity in this study. Old adult serum samples used in this study were described previously [19]. The other group (N = 55) was from young adults (<42 years of age) who were immunized with PPV23 one month before phlebotomy. Young adult serum samples were obtained from M. Blake (Bethesda, MD) and described in our previous study [20].

### Inhibition ELISA

Inhibition ELISA was performed as described in 3^rd^ generation pneumococcal antibody ELISA (www.vaccine.uab.edu) with some modifications. Wells of medium-binding microtiter plates were coated at 37°C with a pre-determined concentration (10 µg/ml) of capsular PS serotype 23F (ATCC, Rockville, MD) for 5 hr in phosphate buffered saline (PBS) with 0.02% NaN_3_. Serum samples (diluted 1∶200) were pre-absorbed with 5 µg/ml of cell wall polysaccharide (C-PS) and 5 µg/ml of serotype 22F PS (ATCC, Rockville, MD). Human sera were then incubated in duplicate for one hour at room temperature in the presence of different amounts of inhibitors (L-rhamnose or pneumococcal serotype 23F PS). The plates were washed with PBS containing 0.05% Tween-20 (PBS-T) and then loaded with alkaline phosphatase (AP)-conjugated goat antibody specific for human IgG in PBS-T with 0.02% NaN_3_. After 2 hr of incubation at RT, the plates were washed. The amount of enzyme conjugate immobilized to each well was determined with the addition of the *p*-nitrophenyl phosphate substrate (Sigma, St. Louis, MO) in diethanolamine buffer and incubation at room temperature for 2 hr. The reactions were stopped with NaOH, and the optical density at 405 nm was measured using an ELISA microplate reader. The percentage of inhibition was calculated as follows: percent inhibition  =  [1-(absorbance of serum with inhibitor/absorbance of serum without inhibitor)] X 100%.

### Inhibition opsonophagocytic killing assay (OPA)

Inhibition OPA was performed for serotypes 14 and 23F as described previously with modifications [21]. Briefly, pneumococci expressing serotypes 14 and 23F had been made to be resistant to streptomycin and trimethoprim, respectively, but susceptible to the other antibiotics. Frozen aliquots of target pneumococci were thawed, washed twice with Opsonization Buffer (Hanks' balanced salt solution (HBSS) with Mg/Ca, 0.1% gelatin, and 10% fetal bovine serum) by centrifugation (12,000×g, 2 minutes), and diluted to the proper bacterial density (∼2×10^5^ cfu/ml of each serotype). Equal volumes of two bacterial suspensions were pooled. All serum samples were incubated at 56°C for 30 minutes before serial dilutions in Opsonization Buffer. Serially diluted serum (20 µl/well) was added in the presence inhibitors (L-rhamnose, L-fucose, D-galactose or pneumococcal serotype 23F PS) and incubated at room temperature for 30 minutes with shaking at 700 rpm. Diluted serum (20 µl/well) was mixed with 10 µl of bacterial suspension in each well of round bottom 96-well plates (Corning Inc., Corning, NY). After 30 minute incubation at RT with shaking at 700 rpm, 10 µl of 3–4 week-old-rabbit complement (PelFreeze Biologicals, Rogers, Arkansas) and 40 µl of differentiated HL60 cells (4×10^5^ cells) were added to each well. HL60 cells were differentiated to granulocytes by culturing in RPMI-1640 with 10% fetal bovine serum and 1% L-glutamine and 0.8% dimethylformamide at a starting density of 4×10^5^ cells/ml for 5–6 days. Plates containing the bacteria, antibody and HL60 cells were incubated in a tissue culture incubator (37°C, 5% CO_2_) with shaking at 700 rpm. After 45 minute incubation, plates were placed on ice for 10–15 minutes and an aliquot of the final reaction mixture (10 µl) was spotted onto 3 different THY agar plates (Todd-Hewitt broth with 0.5% yeast extract and 1.5% agar). When the fluid was absorbed into the agar, an equal volume of an overlay agar (THY with 0.75% agar and 25 mg/L of TTC) containing one of the two antibiotics was applied to each THY agar plate. After an overnight incubation at 37°C, the number of bacterial colonies in the agar plates was enumerated. Opsonization titers were defined as the serum dilution that kills 50% of bacteria. A detailed protocol is posted on our website (www.vaccine.uab.edu).

### Affinity chromatography for absorption of IgM antibodies from immune sera

Absorption of IgM antibodies from human immune sera was performed as previously described [20]. To remove isotype IgM antibodies from immune sera, 10 µl of each serum sample was mixed with 90 µl of agarose beads (25% slurry) conjugated with antibody specific for anti-human IgM (Sigma, St. Louis, MO) for 2 hrs at RT. The mixture was then passed through a Bio-Rad chromatography spin column (Bio-Rad Laboratories, Inc., Hercules, CA) by centrifugation at 2,000 rpm for 1 min at 4°C. About 75 µl of sample was obtained after centrifugation. For mock absorption, unconjugated agarose beads were used instead of antibody conjugated beads. The final antibody level of each isotype was measured by 3^rd^ generation pneumococcal antibody ELISA in order to monitor the completeness of IgM absorption [22].

### Statistical analysis

Young and old adults were compared using Fisher's exact test. P-values less than 0.05 were considered to be significant. Statistical calculations were performed with use of JMP software 8.0 (SAS Institute Inc.).

## Results

### Specificity of anti-pneumococcal serotype 23F PS antibody for L-rhamnose

In order to determine if L-rhamnose is part of epitope recognized by human serum antibodies induced with PPV23, binding of serum antibodies to pneumococcal serotype 23F PS was inhibited by various concentrations of L-rhamnose using ELISA. We randomly selected six immune sera (S1–S6) from old adults whose IgG concentrations were in low (S5 and S6), middle (S3), or high (S1, S2 and S4) groups from our previous study [19]. The concentrations of IgG, IgM and opsonic indices of each serum sample are shown in Table 1. Among six immune sera, four sera showed 50% inhibition by approximately 0.05–10 mM of L-rhamnose. In particular, sample 5 (S5) showed the highest affinity, where 50% inhibition of serum antibody binding to PS was achieved at 0.04 mM of L-rhamnose (Table 1).

**Table 1:** Concentrations and opsonic indices of serum antibodies against pneumococcal PS serotype 23F.

	Antibody concentrations (ug/ml)			
Sample ID^a^	IgG^c^	IgM^d^	IC50^e^ (mM)	Opsonic index
S1	26.75	0.16	5.91	11,268
S2	39.59	0.35	0.08	4,538
S3	5.67	0.07	0.31	1,594
S4	21.64	0.26	NI^f^	3,664
S5	2.41	ND^b^	0.04	666
S6	1.57	0.10	NI^f^	1,922

^a^ Serum samples were obtained from old adults immunized with PPV23.

^b^ ND: Not determined.

^c,d^ Antibody concentrations were determined by 3^rd^ generation pneumococcal antibody ELISA methods and a computer program for pneumococcal antibody ELISA (CDC program). (www.vaccine.uab.edu).

^e^ 50% Inhibition Concentration (IC50) indicates concentration of L-rhamnose that requires 50% of serum antibody binding to polysaccharide coated on 96-wells microtiter plate.

^f^ NI: Not inhibited.

### Inhibition of opsonophagocytic killing of *S. pneumoniae* serotype 23F by L-rhamnose

**Figure 2:**
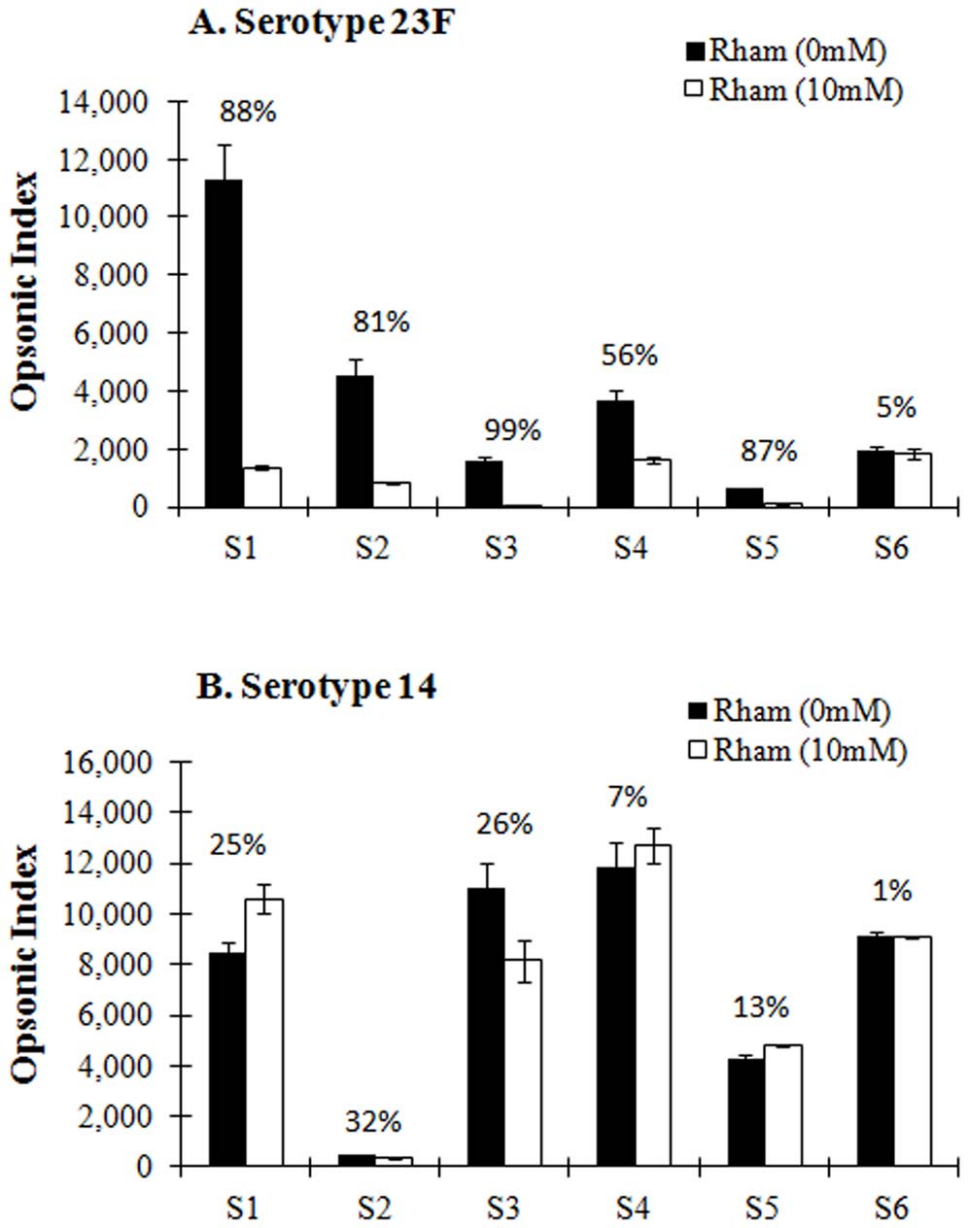
Changes in opsonic index after inhibition by rhamnose. Opsonic capacities of serum samples obtained from adults (S1–S6) immunized with PPV23 were measured in the presence or absence of 10 mM L-rhamnose against pneumococcal serotype 23F (A) or 14 (B). Each serum sample is shown as S1, S2, S3, S4, S5 and S6. The numbers (%) indicate the percent change of opsonic indices in the presence of 10 mM L-rhamnose. Data shown are the mean ± standard error (SE) of two independent experiments.

To study the opsonic capacities of the six immune sera from old adults, they were evaluated with *in vitro* OPA with or without 10 mM L-rhamnose. 10 mM rhamnose was chosen since it was sufficient to inhibit the binding of the antibodies in the sera (Table 1). Among six immune sera, four sera (S1, S2, S3, and S5) showed more than 80% of inhibition in opsonic indices, whereas only one (S6) of the immune sera was not inhibited by 10 m M L-rhamnose (Figure 2A). No significant non-specific killing was detected when we evaluated the irrelevant pneumococcal serotype 14 (Figure 2B). Only S2 showed more than 30% inhibition, which is probably not rhamnose-specific inhibition considering its low opsonic capacity. The capacity of other relevant sugars to inhibit pneumococcal serotype 23F bacteria such as L-fucose and D-galactose was also tested and they showed no effect on bacterial killing at the concentration of 50 mM for each sugar (data not shown). The effector cells (differentiated HL-60 cells) appeared to be unchanged under microscopy in the presence of 10 mM of sugars suggesting their membranes were not disrupted (data not shown). We extended this observation by studying many serum samples from young (N = 31) or old adults (N = 22) immunized with PPV23. In 36% of immune sera, opsonic capacity was inhibited more than 60% by 10 mM L-rhamnose, and 30% of immune sera showed 30–60% inhibition of opsonic index (Table 2). Thus, L-rhamnose is most likely a part of immunodominant epitope for functional antibodies specific for serotype 23F PS. 16 pre-immune sera from old adults were also analyzed using OPA in order to determine anti-23F PS antibodies are induced by PPV23 vaccination, not by natural exposure or previous PPV23 vaccination. Their geometric mean indices (GMI) was 264, which is five folds less than GMI of post-immune sera (GMI = 1,289). Moreover, in the presence of 10 mM L-rhamnose, about 70% of opsonic indices (GMI) are inhibited in both pre- and post-immune sera (data not shown).

**Table 2:** Number of serum samples with 23F PS specific antibodies which are inhibited by 10-rhamnose in OPKA before- or after-IgM antibody depletion.

	No. of serum samples showing inhibition by 10 mM L-rhamnose					
	Before-IgM depletion^b^			After-IgM depletion^c^		
Age^a^	<30% inhibition	30–60% inhibition	>60% inhibition	<30% inhibition	30–60% inhibition	>60% inhibition
Old (N = 22)	8 (36%)	6 (27%)	8 (36%)	8 (36%)	6 (27%)	8 (36%)
Young (N = 31)	10 (32%)	10 (32%)	11 (33%)	7 (23%)	3 (10%)	21 (68%)
Total	18	16	19	15	9	29

^a^ Age: old adults (70–79 yrs), young adults (<42 yrs)

^b,c^ Fisher's exact test was performed to compare old and young adults (*p*-value = 0.917 and 0.029 for each comparison of before-^b^ and after-IgM^c^ depletion, respectively).

### Comparison of rhamnose specific serum antibodies between young and old adults

In order to investigate the difference in antibody repertoire with aging, we examined epitope specificity of serotype 23F PS specific antibodies by comparing the rhamnose inhibitable antibody pattern between young and old adults immunized with PPV23, since branched sugars in pneumococcal polysaccharide are known to be immunogenic [16]. The serum samples are divided into groups of inhibition ratios of less than 30%, 30–60% and more than 60%, and shown in Table 2. There was no significant difference between young and old adults (*p*-value = 0.917, Fisher's exact test).

### Comparison of rhamnose specific IgG antibodies in the sera between young and old adults

**Figure 3:**
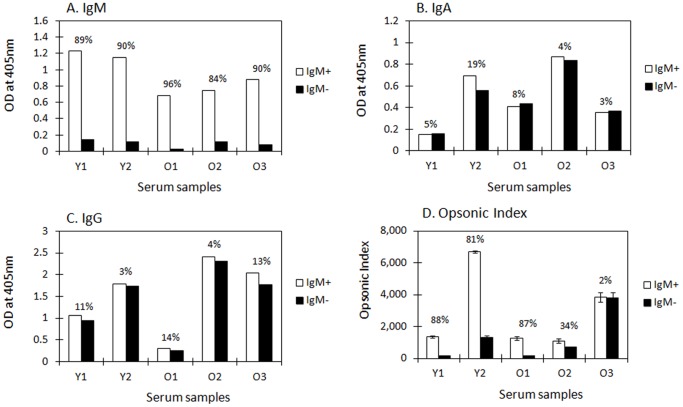
Absorption of IgM from sera immunized with PPV23. Five representative serum samples (Y1, Y2, O1, O2 and O3) were randomly selected from young (Y) or old adults (O) immunized with PPV23. The levels of IgA and IgM were measured at 80-fold dilution and the level of IgG was measured at 400-fold dilution. IgM+ and IgM- indicate mock-absorption and IgM-absorption, respectively. The levels of serotype 23F specific IgM (A), IgA (B), and IgG (C) are shown for mock- or IgM-absorption from immune sera. We chose 80-fold dilution for IgA and IgM, and 400-fold dilution for IgG based on a serial dilution curve ELISA. The numbers (%) indicate the percent change in OD at 405 nm (A, B, C) or OI after IgM absorption. D, Opsonic index (OI) of five immune sera is shown after mock- or IgM-absorption. Data shown in Figure 3D are the mean ± standard error (SE) of two independent experiments.

We did not observe a significant difference between young and old adults when we analyzed the difference in the proportion of L-rhamnose inhibitable antibodies with whole serum. Since IgM antibodies are opsonic and we observed in our previous study that young adults have more IgM antibodies than old adults [20], studies of the whole serum do not permit the comparison of L-rhamnose specific IgG antibodies. Therefore, we depleted IgM antibodies from immune sera by affinity chromatography to study the specificity of anti-23F PS IgG antibodies for L-rhamnose. 53 serum samples from both young and old adults that showed more than 80% of IgM absorption and less than 20% changes in IgA or IgG antibody levels after affinity chromatography were randomly chosen for *in vitro* OPA. Figure 3 and Table 3 show five representative samples among immune sera from young and old adults. Opsonic indices were decreased by more than 50% in serum samples from 2 young adults (Y1 and Y2) and one of old adults (O3) (Figure 3D).

**Table 3:** Concentrations and opsonic indices of serum antibodies against pneumococcal PS serotype 23F.

	Antibody concentrations (ug/ml)		Opsonic index	
Sample ID^a^	IgG^b^	IgM^c^	Before IgM depletion	After IgM depletion
Y1	3.67	0.37	1,341	155
Y2	9.01	0.31	6,696	1,298
O1	0.93	0.09	1,273	168
O2	8.45	0.11	1,104	724
O3	9.46	0.12	3,855	3,785

^a^ Serum samples were selected randomly from young or old adults immunized with PPV23 which show more than 80% depletion of IgM and less than 20% depletion of IgA and IgG after affinity chromatography.

^b,c^ Antibody concentrations were determined by 3^rd^ generation pneumococcal antibody ELISA methods and computer program for pneumococcal antibody ELISA (CDC program) (www.vaccine.uab.edu).

Next, we analyzed the L-rhamnose inhibition pattern with IgM antibody-depleted serum samples in order to define L-rhamnose inhibitable IgG antibodies. We compared the proportion of serum samples whose opsonic capacities were highly inhibited (>60%) by 10 mM of L-rhamnose before and after IgM depletion. The number of serum samples with high inhibition increased from 11 (33%) before IgM depletion to 21 (68%) in young adults, but did not change in old adults (*p*-value  = 0.029; Fisher's exact test) (Table 2).

## Discussion

The pneumococcal serotype 23F PS repeating unit has two L-rhamnose residue, one is in the backbone (β(1->4)-linked L-rhamnose) and the other is a branch from the repeating unit backbone (α(1->2)-linked L-rhamnose) (Figure 1) [5]. According to previous studies with rabbit and horse antisera against pneumococcal serotype 23F PS, a carbohydrate structure with branched residue α(1->2)-linked L-rhamnose is most likely the immunodominant epitope [16]. Since terminal nonreducing sugars were found to be antigenic determinants of PS antigens in many examples, we assume that the branched residue, α(1->2)-linked L-rhamnose, is a possible dominant epitope of pneumococcal serotype 23F PS for humans. Reason *et al*. also reported epitope specificity of a pneumococcal serotype 23F PS-specific Fab for the hapten L-rhamnose using a recombinant Fab library isolated from humans immunized with PCV7 or PPV23 [23], [24]. In addition, synthetic tri- and tetra-saccharides, which were synthesized based on the structure of pneumococcal serotype 23F PS and contained L-rhamnose, were demonstrated to be immunogenic and protective against pneumococcal infection in animals [16]. Our analysis of L-rhamnose specific serum antibodies against serotype 23F PS using functional assays concurs with these previous studies and also reveals that a significant proportion of subjects (19/53) have L-rhamnose inhibitable antibodies.

*S. pneumoniae* commonly colonize in respiratory tract of normal individuals and the antibodies in the sera may have been induced by colonization, not by the vaccine. Also, old adults in this study had been vaccinated with PPV23 >5 years prior. Thus, antibodies in the post-immunization sera, particularly those from old adults, may have been induced by the natural colonization or the previous PPV23. We have therefore analyzed pre-immune sera from 16 old adults using rhamnose inhibition OPK assay. Five folds lower GMI of 16 pre-immune sera (GMI = 264) from old adults than GMI of post-immune sera (GMI = 1,289), and their inhibition in the presence of 10 mM L-rhamnose suggest that rhamnose specific antibodies observed in the post-immune sera of this study are most likely induced by PPV23 vaccination, not by natural exposure to pneumococci or previous PPV23. This conclusion is also consistent with a recent study, which concluded that vaccine naïve and vaccine-experienced adults (>5 year prior) show no difference in 23F PS specific IgG antibody concentration, opsonic activity and B-cell phenotype distribution [25].

PPV23 can also induce pneumococcal PS specific IgM or IgA antibodies besides IgG, and those antibodies were reported to be functional. In particular, IgM antibodies are very efficient in complement fixation which may explain the high opsonic capacity of IgM antibodies [20], [26], [27]. In order to preferentially study the L-rhamnose specificity of IgG antibodies, we depleted IgM antibodies from immune sera and performed OPA. Interestingly, after depletion of IgM antibodies from immune sera, the proportion of serum samples, which showed an L-rhamnose inhibitable antibody pattern, increased in young adults but not in old adults, suggesting that the serum IgG antibody repertoire represented in old adults may be different from that of young adults. Nevertheless, it is difficult to define the age-related diversity of the antibody repertoire with only L-rhamnose inhibitable pattern. For instance, some serotype 23F PS specific serum antibodies showed no inhibition pattern both in ELISA and OPA in the presence of 10 mM of rhamnose in our study (e.g. Sample 6 in Figure 3 and Table 1) indicating that rhamnose is not the only epitope which induces opsonic antibodies. Therefore, future study will have to elucidate the antibody repertoire difference in more detail.

Age-associated changes include a decrease in the activity of B cell class switching recombination (CSR) and somatic hypermutation (SHM), which are known to have effects on antibody affinity, avidity, and isotype [28], [29]. Previous studies also found shifts in antibody variable light chain gene usage upon aging [30]. However, some studies reported that the variable region repertoire is maintained with advancing age [31], [32]. Therefore, it is still unclear whether the antibody repertoire changes with aging in humans.

Synthetic tri- and tetra-saccharides, which were synthesized based on the structure of pneumococcal serotype 23F PS and contained L-rhamnose, were demonstrated to be immunogenic and protective against pneumococcal infection in animals [16]. Synthetic carbohydrate-based vaccines were also studied as vaccine candidates against other species of encapsulated bacteria, e.g. *S. aureus*, *H. influenzae* [33]–[35]. But animal studies provide unreliable guidance since the immunogenicity of the chemical constructs is species dependent. This limitation may be overcome if various chemical constructs can first be tested for their ability to bind to human immune sera. As we demonstrated with many human sera obtained from subjects immunized with PPV23 in this study, the rhamnose targeting anti-23F antibodies are opsonic, and, therefore, rhamnose may be a target for developing a chemically defined epitope for pneumococcal vaccines.

After the introduction of PCV7 to protect young children from pneumococcal diseases, the prevalence of nasopharyngeal carriage of non-vaccine serotype bacteria has increased [36]–[39]. To broaden the serotypic coverage, PCVs need to include more PSs from previously non-vaccine pneumococcal serotypes. However, due to the technical limitations of the conjugation of PS and carrier proteins in conjugate vaccines, simple carbohydrate structures which can elicit antibodies against pneumococci have been studied extensively as vaccine targets [12]–[15]. Considering the high immunogenicity in a significant number of individuals immunized with PPV23, the carbohydrate structure with L-rhamnose could be useful for future vaccine design.
