# Correction: L-Rhamnose Is Often an Important Part of Immunodominant Epitope for Pneumococcal Serotype 23F Polysaccharide Antibodies in Human Sera Immunized with PPV23

**DOI:** 10.1371/annotation/84458b5b-2bae-4d71-b003-7d584c1bfd8c

**Published:** 2014-01-13

**Authors:** Saeyoung Park, Moon H. Nahm

In the Author Contributions section, Moon H. Nahm (MHN) should be listed as one of the persons who contributed reagents/analysis/tools and as one of the persons who wrote the paper.

